# Non-native proteins inhibit the ER oxidoreductin 1 (Ero1)–protein disulfide-isomerase relay when protein folding capacity is exceeded

**DOI:** 10.1074/jbc.RA119.011766

**Published:** 2020-02-26

**Authors:** Antti Moilanen, Lloyd W. Ruddock

**Affiliations:** Faculty of Biochemistry and Molecular Medicine, University of Oulu, 90220 Oulu, Finland

**Keywords:** disulfide, unfolded protein response (UPR), protein disulfide-isomerase, protein misfolding, endoplasmic-reticulum-associated protein degradation (ERAD), ER oxidoreductin (Ero1), feedback inhibition, hyperoxidation, oxidative folding, regulation

## Abstract

Protein maturation in the endoplasmic reticulum (ER) depends on a fine balance between oxidative protein folding and quality control mechanisms, which together ensure high-capacity export of properly folded proteins from the ER. Oxidative protein folding needs to be regulated to avoid hyperoxidation. The folding capacity of the ER is regulated by the unfolded protein response (UPR) and ER-associated degradation (ERAD). The UPR is triggered by unfolded protein stress and leads to up-regulation of cellular components such as chaperones and folding catalysts. These components relieve stress by increasing folding capacity and up-regulating ERAD components that remove non-native proteins. Although oxidative protein folding and the UPR/ERAD pathways each are well-understood, very little is known about any direct cross-talk between them. In this study, we carried out comprehensive *in vitro* activity and binding assays, indicating that the oxidative protein folding relay formed by ER oxidoreductin 1 (Ero1), and protein disulfide-isomerase can be inactivated by a feedback inhibition mechanism involving unfolded proteins and folding intermediates when their levels exceed the folding capacity of the system. This mechanism allows client proteins to remain mainly in the reduced state and thereby minimizes potential futile oxidation–reduction cycles and may also enhance ERAD, which requires reduced protein substrates. Relief from excess levels of non-native proteins by increasing the levels of folding factors removed the feedback inhibition. These results reveal regulatory cross-talk between the oxidative protein folding and UPR and ERAD pathways.

## Introduction

Oxidative protein folding in the endoplasmic reticulum (ER)^2^ is tightly connected to quality control mechanisms to avoid the accumulation of misfolded proteins that would lead to cell stress. First, the formation of disulfide bonds is subjected to tight regulation to avoid hyperoxidation of the ER (1–3). Second, in the event that the load of unfolded proteins exceeds the folding capacity of the ER, signaling pathways are activated to restore the balance (4). These are collectively termed the unfolded protein response (UPR). Two parts of the UPR are to increase the folding capacity of the ER by up-regulating folding factors and to remove non-native proteins by up-regulation of ER-associated degradation (ERAD) components.

Under steady-state conditions in the mammalian ER, disulfides are formed mainly by the action of sulfhydryl oxidases of the ER oxidoreductin 1 (Ero1) family (two isoforms in human, Ero1α and Ero1β), which reduce molecular oxygen to hydrogen peroxide in the process (5–7). The newly formed disulfides are then transferred to members of the protein disulfide-isomerase (PDI) family, which in turn oxidize client proteins. The canonical PDI has four domains, **a**, **b**, **b′**, and **a′**, with **a** and **a′** containing the Cys-Xaa-Xaa-Cys active sites that catalyze thiol-disulfide exchange reactions and **b′** forming the principle substrate-binding site. Both active sites have been shown to exist as a mixture of oxidized and reduced species *in vivo* (8). Although the oxidized PDI pool carries out oxidation of nascent polypeptides, the reduced pool is crucial for isomerization reactions correcting non-native disulfides in misfolded proteins (9). Therefore, a balance between the oxidized and reduced state of PDI needs to be retained. One way to avoid hyperoxidation of PDI and the ER is to inactivate Ero1 under oxidizing conditions by rearrangement of its regulatory disulfides, which effectively shuts down access to the Ero1 active site (1, 10). Once redox homeostasis is restored, Ero1 can be reactivated by reduced PDI (2). Both processes, the catalytic oxidation of PDI as well as the activation steps, have been suggested to be mediated by the same molecular determinant: a protruding β-hairpin with a tryptophan at the tip (Trp^272^) in Ero1 that binds to the **b′** substrate-binding site of PDI (11, 12).

The mammalian UPR is a complex signaling network that results in measures to restore protein-folding homeostasis by attenuation of protein synthesis, transcriptional up-regulation of chaperones, and folding catalysts (13) and by increasing ERAD (14). In contrast to the other aspects of the UPR, which aim to enhance productive oxidative protein folding, ERAD requires reduction of disulfides and unfolding of the client proteins for the translocation processes (15). These reductive processes are generally thought to be catalyzed by PDI family members such as PDI and ERdj5 (16–18).

The cross-talk between oxidative protein folding and UPR-ERAD pathways is not completely understood. In particular, it may be productive to modulate oxidative rates under some circumstances to reduce the burden for reductive processes, which could positively influence ERAD. We are unaware of any direct biochemical evidence that shows how this redox modulation could be achieved.

Here we show that the activity of Ero1α, especially the redox activation steps, is strongly inhibited by unfolded proteins or other PDI ligands when their levels surpass that of PDI. Furthermore, active Ero1α may undergo redox inactivation during this inhibition. Restoring the levels of PDI to exceed those of its ligands allows activation of Ero1α permitting oxidative folding to proceed. These results indicate that the Ero1α–PDI pathway is initially inactivated when subjected to a high load of non-native proteins. This novel regulatory mechanism allows client proteins to remain mainly in the reduced state preventing futile oxidation–reduction cycles if the clients are destined for ERAD. A UPR-based increase in the levels of folding factors or removal of unfolded protein by ERAD then overcomes the inhibitory feedback mechanism allowing oxidative folding.

## Results

### Non-native proteins inhibit Ero1α activity and activation

**Figure 1.**
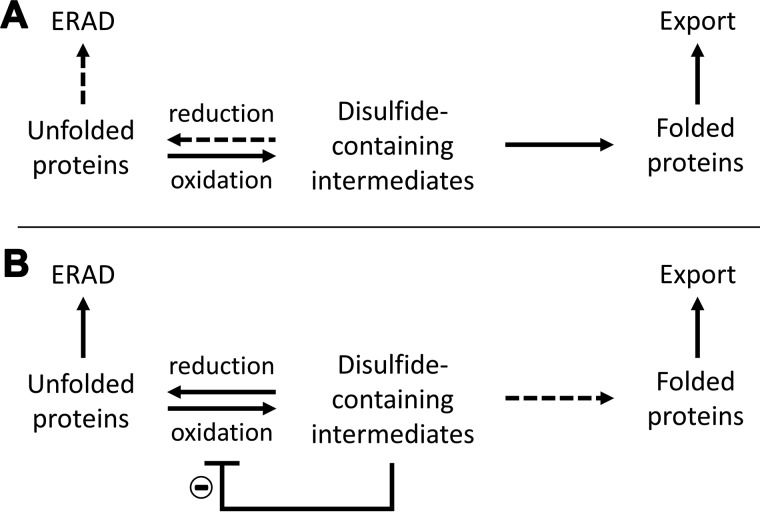
**Schematic for potential feedback inhibition.** *A*, in normal conditions, the concentration of disulfide-containing folding intermediates is low, and there is free PDI to keep the Ero1α–PDI relay active. Folding intermediates will fold to their native state and will be exported from the ER. In this situation, there is minimal need for the reducing pathways leading to ERAD. *B*, in stress situations, the concentration of the folding intermediates increases, and the folding capacity of the cell may be exceeded, leading to reduced export, accumulation of folding intermediates, and an increased need for reductive pathways leading to ERAD. A feedback inhibition loop, based on the sequestration of PDI by binding non-native proteins, will inhibit oxidative folding. Such feedback inhibition will prevent futile oxidation–reduction cycles and keep the folding intermediates in a more reduced state, making them more amenable for ERAD.

We hypothesized that it may be beneficial to inhibit disulfide bond formation when the load from unfolded or non-native proteins overcomes the productive protein-folding capacity. This would prevent potential futile redox cycles and would lessen the reductive burden for ERAD (Fig. 1). Human Ero1α requires high concentrations of reduced substrate PDI for redox activation, as well as for catalytic turnover (12). Both events are mediated by the same molecular determinant, specifically, the protruding β-hairpin of Ero1α interacting with the hydrophobic substrate-binding site of PDI in the **b′** domain (11, 12). Non-native proteins, such as misfolded proteins or folding intermediates, contain exposed hydrophobic amino acids that are bound by the same substrate-binding site in the **b′** domain of PDI (19) that interacts with Ero1α. This binding is independent of whether the non-native protein contains free thiols or disulfides. Together, these two binding events using the same site in PDI provide a potential mechanism for a feedback inhibition loop: when the non-native proteins are in excess over PDI, there would be no free PDI available to interact with Ero1α either for mediating its activation or for its catalytic cycle.

**Figure 2.**
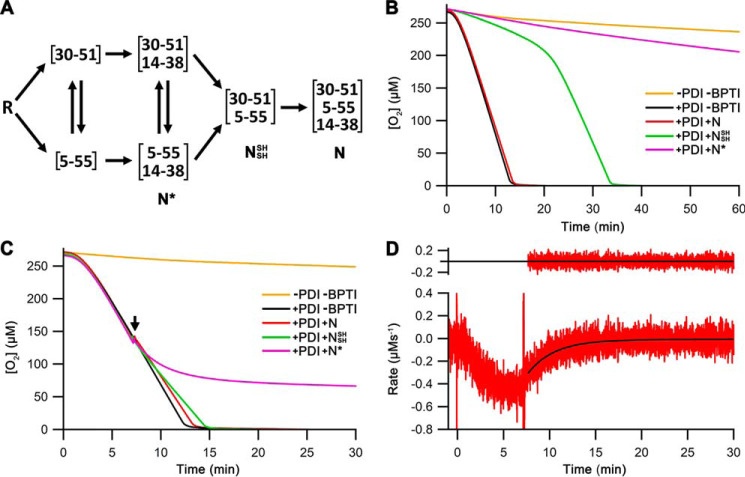
**Oxygen consumption assays of Ero1α–PDI, including late folding species of BPTI as inhibitors.** *A*, schematic of the BPTI folding pathways from the reduced state (*R*) to the native state (*N*) through folding intermediates including the two-disulfide states N* and N_SH_^SH^ (based on Ref. 20). *B*, representative oxygen consumption traces for Ero1α–PDI ± 50 μm BPTI variants as indicated. *C*, representative oxygen consumption traces for Ero1α–PDI as in *B*, but the BPTI variants were injected at ∼50% [O_2_] (marked by an *arrow*) as indicated. *D*, representative kinetic profile for redox inactivation of Ero1α induced by N*. The oxygen consumption trace [+PDI +N*] from Fig. 2*C* was processed to give a plot of oxygen consumption rate *versus* time (12). These data were used to obtain a one-step exponential fit (*black fit*) for the region after the injection (marked with an *arrow*) of N*. The first 20-s postinjection was excluded from the analysis because of mixing artifacts. *Upper panel*, residuals for the fit.

To test the hypothesis, we carried out oxygen consumption–based activity assays of Ero1α–PDI in the presence or absence of different variants of bovine pancreatic trypsin inhibitor (BPTI), a small protein with a well-characterized oxidative folding pathway (Fig. 2*A*) and three disulfides (Cys^5^–Cys^55^, Cys^14^–Cys^38^, and Cys^30^–Cys^51^) in the native state (denoted as N) (20).

Addition of N from the start of the reaction had no effect on oxygen consumption by Ero1α–PDI (Fig. 2*B*). This suggested, as expected, that natively folded proteins do not inhibit the activation or the catalytic cycle of Ero1α because they interact with neither Ero1α nor PDI.

We then tested the potential inhibitory effects of two BPTI mutants: C14A/C38A and C30A/C51A. These mimic late folding intermediates N_SH_^SH^ and N*, respectively, in the BPTI folding pathway (Fig. 2*A*). They are incapable of folding to the native three-disulfide state, because they lack a pair of cysteine residues. Both, N_SH_^SH^ and N* compromised the activation of Ero1α, leading to a much longer lag phase (Fig. 2*B*). However, the two folding intermediates showed stark differences at longer time points. Although the inhibition by N* was potent and continued for the duration of the oxygen consumption measurements, N_SH_^SH^ lost its inhibitory effect during the experiments and allowed Ero1α to sharply transit to a completely active state. This implies that N_SH_^SH^ does not inhibit the Ero1–PDI catalytic cycle but that it does inhibit Ero1α activation steps.

To separate activation effects from catalytic cycle inhibitory effects, postactivation assays were performed. Now, the potential inhibitor was added after Ero1α had fully activated. These results (Fig. 2*C*) confirmed that N and N_SH_^SH^ have negligible inhibitory activity toward the Ero1α–PDI catalytic cycle. In contrast, addition of N* resulted in a loss of Ero1α activity. Analyzing the kinetic profile of this region revealed an exponential loss of activity with a half-time of inactivation of ∼128 s (Fig. 2*D*; *n* = 3), a value very similar to the activation half-time of the uninhibited reaction (126 ± 7 s; *n* = 5; see also Ref. 12).

### PDI-mediated activation and catalysis of Ero1 are inhibited by peptides

Although we have no evidence that transient mixed disulfides are formed between PDI and the N_SH_^SH^ or N* mimics of late-stage folding intermediates, it is possible that they do, and the inhibition arises, at least in part, from this. In addition, the inhibition of Ero1α activation and Ero1α–PDI catalysis by BPTI folding intermediates is only semiquantitative because it is impossible to ensure that the redox state of the folding intermediate is not in dynamic exchange in the assay conditions that contain both an oxidase (Ero1α–PDI) and a reductant (reduced GSH). To avoid this issue, we switched to model the inhibition using a peptide that contains no cysteine residues and mimics the exposed hydrophobic core of protein substrates. We chose KFWWFS as the model peptide, because it is sufficiently soluble in aqueous solutions, is easily quantified, is redox insensitive, and binds to the substrate-binding site of PDI (21).

**Figure 3.**
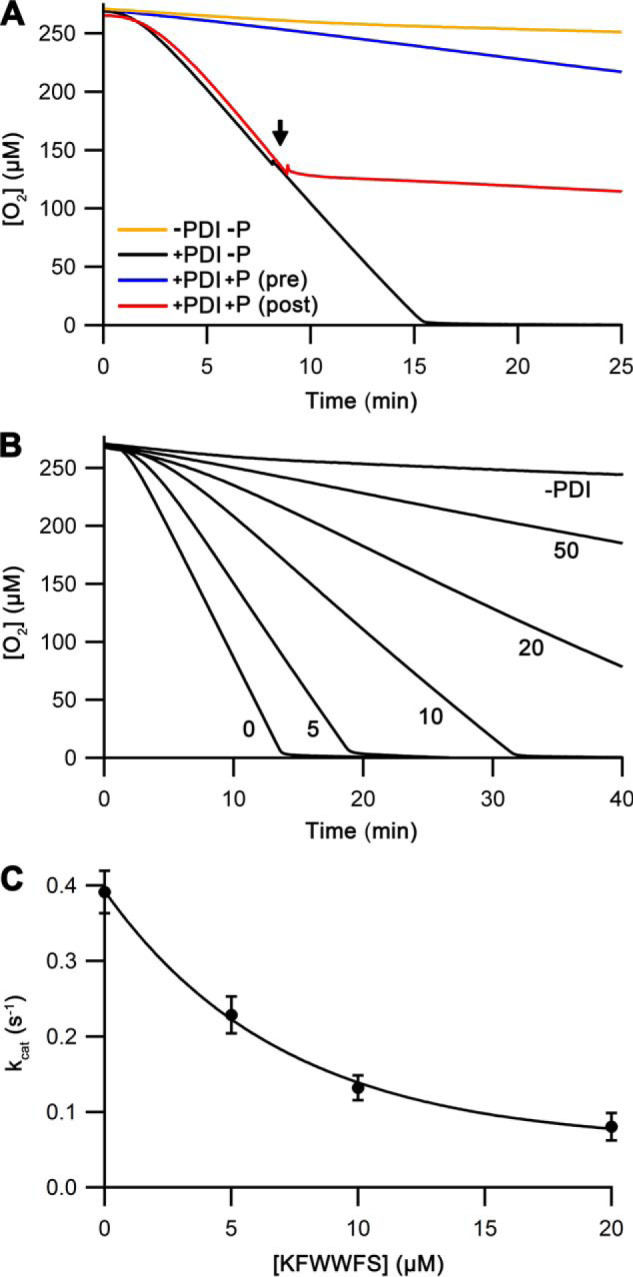
**Quantification of the inhibition using the peptide KFWWFS.** *A*, representative oxygen consumption traces for Ero1α–PDI with or without 50 μm peptide KFWWFS (marked as *P*). The peptide was either premixed (marked *pre*) or injected at ∼50% [O_2_] (marked *post*). The injection point of the peptide or the storage solution only for the control reaction is marked by an *arrow. B*, representative oxygen consumption traces for Ero1α–PDI with varying [KFWWFS] (μm) as indicated. *C*, a plot of *k*_cat_ values (*n* = 3–5; mean ± S.D.) *versus* [KFWWFS] with an exponential fit.

Adding KFWWFS to the assays resulted in an almost complete loss of Ero1α activity irrespective of whether the addition was pre- or postactivation (Fig. 3*A*). This again underlines the importance of the substrate-binding pocket in the **b′** domain of PDI in both Ero1α activation and the catalytic cycle and, importantly, demonstrates that the inactivation is independent of formation of a mixed disulfide between PDI and the inhibitor. To quantify the inhibition, we repeated the preactivation assays but now varied the concentration of the peptide. The results showed a dose-dependent extension of the lag phase and inhibition of the oxygen consumption rate (Fig. 3*B*). Determination of the catalytic rate constant (*k*_cat_) revealed a IC_50_ (*K_i_*) of 4.9 ± 0.6 μm (Fig. 3*C*), but no changes in the *K_m_* (3.8 ± 1.1 μm) or the Hill coefficient (4.4 ± 0.5) for oxygen.

**Figure 4.**
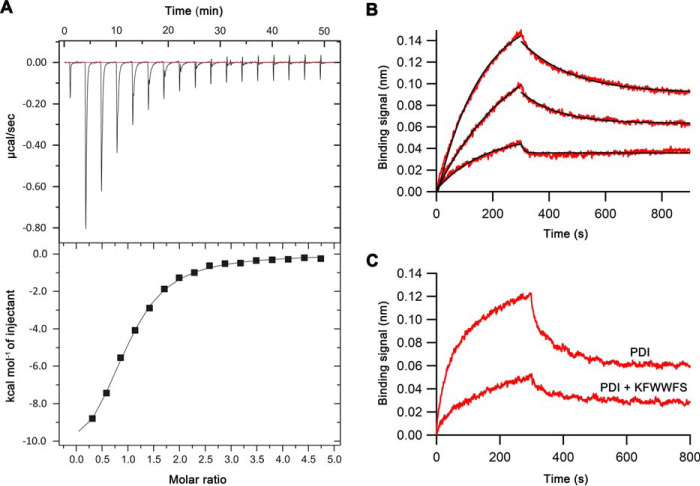
**KFWWFS binding by PDI.** *A*, isothermal titration calorimetry profiles of KFWWFS binding to PDI. The *upper panel* represents the titration profile, and the *lower panel* represents integrated data. *B*, representative BLI binding profile of PDI to immobilized KFWWFS. *Traces* from *top* to *bottom* indicate 2, 1, and 0.5 μm [PDI]. Association and dissociation were individually fit using a 1:1 binding model. *C*, representative BLI binding profile of PDI to immobilized β-hairpin of Ero1α in the absence (*upper trace*) and presence (*lower trace*) of 5× excess KFWWFS.

Although these results were consistent with our hypothesis, *K_i_* values need to be compared with the affinity of PDI for KFWWFS. Affinity was determined using calorimetry (Fig. 4*A*) and bilayer interferometry (BLI; Fig. 4*B*). *K_d_* values of the PDI–peptide complex of 6.8 and 12.7 μm, respectively, were obtained. These were similar to the *K_i_* value for Ero1 activity (4.9 μm), suggesting that inhibition is directly linked to binding of the peptide by PDI, which competes with PDI binding to Ero1.

To test this hypothesis, we undertook a direct binding competition assay by BLI. PDI bound to the β-hairpin loop of Ero1α with an affinity of 4.0 μm. Addition of a 5× excess of KFWWFS to PDI greatly reduced the binding of PDI to the immobilized β-hairpin loop of Ero1α (Fig. 4*C*). This confirms direct competition of binding. Taken together, this confirms the observations from folding intermediate studies that the decreased activity of Ero1α is caused by saturation of the substrate-binding site of PDI by the inhibitors leading to a dose-dependent inhibition of both Ero1α activation rate and catalytic efficiency.

### PDI binds small hydrophobic molecules, which inhibit the regulatory and catalytic activity of Ero1α

In addition to interacting with non-native proteins *in vivo*, PDI also binds a variety of small molecules, both endogenous, such as 17β-estradiol (17β-E2) (22), and exogenous, for example, the endocrine disruptor bisphenol A (BPA) (23). It has been shown previously that BPA inhibits the catalytic cycle of Ero1 (24), but its effect on Ero1 activation has not been reported. Because our results suggested that non-native protein and peptide binding inhibited both Ero1α activation and the Ero1α–PDI catalytic cycle, it is plausible that 17β-E2 and BPA might have the same effect.

**Figure 5.**
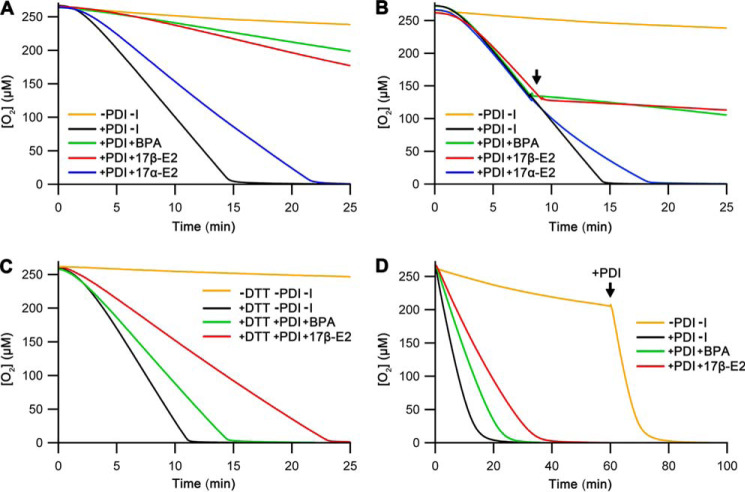
**Inhibition by small hydrophobic molecules.** *A* and *B*, representative oxygen consumption traces with the indicated inhibitors at the start (*A*) or injected at ∼50% [O_2_] (*B*). *I*, inhibitor. *C*, representative oxygen consumption reactions in the absence or presence of the indicated inhibitors were carried out as in *A*, but with an additional 10 mm DTT present at the start where indicated. *D*, representative oxygen consumption traces were collected as in *A* but injecting 1 μm Erv1p instead of the Ero1α–PDI complex. The control reaction without PDI or inhibitor was initiated without exogenous PDI, which was injected at 60 min.

To test this hypothesis, we measured the activity of Ero1α–PDI by following oxygen consumption in the absence or presence of 17β-E2 or BPA. 17α-Estradiol (17α-E2), which has not to our knowledge been reported to be bound efficiently by PDI (25), was used as a control. Both 17β-E2 and BPA strongly compromised oxygen consumption by Ero1α (Fig. 5*A*), indicating that either activation and/or catalytic activity of Ero1α was inhibited by these compounds. When the compounds were added to preactivated Ero1α, both caused a severe inhibition of oxygen consumption (Fig. 5*B*). In contrast, the effects of 17α-E2 in both assays were less severe, causing a slightly prolonged lag phase and slightly decreased maximal rate, *i.e.* it inhibited both activation and catalytic turnover, but only marginally. To rule out that the loss of activity was caused by a direct inhibition of Ero1α, we repeated the assays in the presence of DTT, a thiol compound capable of accepting disulfides from Ero1α. With DTT present, Ero1α regained enzymatic activity in the presence of both strong small molecule inhibitors (Fig. 5*C*), confirming that the inhibitors mainly affected the Ero1α–PDI interaction, which in turn affects both activation of Ero1α and catalytic activity of the enzyme toward its natural substrate PDI.

As an additional control, we measured similar inhibition assays replacing Ero1α with another sulfhydryl oxidase, Erv1p, capable of oxidizing PDI despite lacking the hairpin structure of Ero1α (12, 26). The oxygen consumption rate was not strongly inhibited by either 17β-E2 or BPA (Fig. 5*D*). Because Erv1p showed a negligible oxygen consumption rate in the presence of GSH only and regained complete activity upon injection of PDI (Fig. 5*D*), we conclude that Erv1p bypasses the inhibition seen for Ero1α by acting directly on the active site of PDI without the need to interact with the substrate-binding site in the **b′** domain.

### Relief from excess non-native protein restores Ero1α activation and activity

Any feedback inhibition of protein oxidation would need to be reversible to have physiological relevance. To examine this under controlled conditions, two experiments with different mechanisms for the reversal were undertaken: (i) folding of the substrates and (ii) increase in protein folding factors.

**Figure 6.**
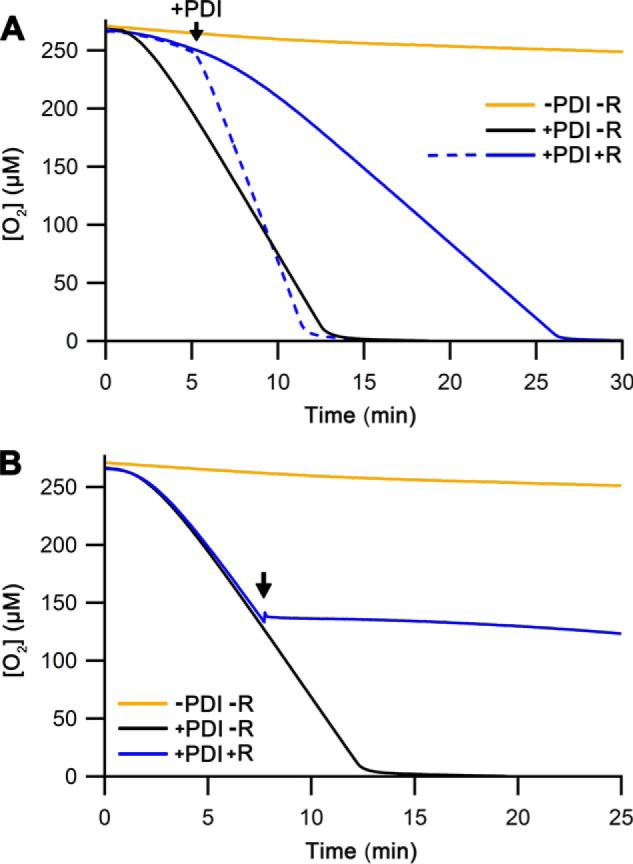
**Relief from excess non-native proteins by increasing the levels of folding factors.** *A*, representative oxygen consumption traces for Ero1α–PDI using 10 μm PDI and 20 μm unfolded reduced BPTI (*R*) at the start of the reaction. The increase in folding catalysts over non-native protein was initiated by injecting (*dashed line*) or not injecting (*solid line*) 20 μm PDI at the point indicated by an *arrow. B*, representative oxygen consumption traces for Ero1α–PDI were collected as in Fig. 2*C* with the exception of injecting reduced BPTI (*R*) instead of the late-folding intermediates at ∼50% [O_2_] (marked by an *arrow*).

First, denaturated and reduced WT BPTI in 2-fold molar excess over PDI was added to the Ero1α activity assay. Reduced BPTI inhibited initial activation of Ero1α, resulting in a delayed lag phase (Fig. 6*A*). WT BPTI can be refolded to the native state, and during the time course of the reaction, the rate of oxygen consumption increased to 31% of the maximal observed in the absence of inhibitors; *i.e.* as non-native proteins are removed from the system, the inhibition is relieved. In a postactivation inhibition assay, reduced BPTI strongly inhibited the catalytic cycle (Fig. 6*B*). This is in stark contrast to the late-stage folding intermediates, which did not markedly inhibit the catalytic cycle and affected mainly the activation steps (Fig. 2, *B* and *C*). These results suggest that although both the early and the late stage folding intermediate species inhibit strongly the activation steps of Ero1α, only the non-native proteins from the early stages of the folding pathways are capable of inhibiting the catalytic cycle, at least for those intermediates tested.

An excess of non-native protein *in vivo* can be cleared by multiple routes, both folding/ERAD, which result in a decrease in non-native protein levels (as tested above) or by increasing the level of protein folding factors, potentially resulting in the folding factors being in excess of substrate proteins. To test the latter route, we repeated the experiment, but after 6 min, additional PDI was added to shift the relative concentration of the folding factor over the non-native proteins. This immediately restored the activity of Ero1α (Fig. 6*A*).

## Discussion

Ero1α has a strong dependence for reduced PDI for both redox activation and for its catalytic cycle, with hydrophobic interactions playing a major role in binding Ero1α to the substrate-binding site in the **b′** domain of PDI (11, 12). This suggested that other molecules capable of binding to the substrate-binding site of PDI, including non-native proteins, might compete with Ero1α possibly inhibiting both the activation steps and the catalytic cycle. This could give a potential mechanism to prevent futile cycles of oxidation–reduction and reduce the reductive load for ERAD when non-native proteins are in excess, *i.e.* conditions when the UPR is induced.

**Figure 7.**
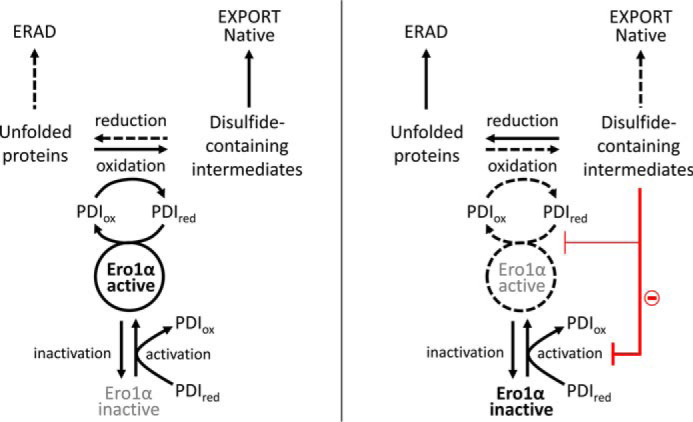
**Schematic for the cross-talk between oxidative protein folding and ERAD.** The *left panel* shows the situation at basal protein synthesis levels when the folding capacity exceeds the burden from newly synthesized non-native proteins. PDI in this situation exists as a mixture of free oxidized and free reduced species (9) and can both oxidize and isomerize client proteins to their native state and also activate any inactivated Ero1α to maintain oxidative protein folding. There is low need for ERAD and associated reducing pathways because the levels of non-native proteins are low because of efficient maturation and export from the ER in the native state. When the folding load increases to exceed the folding capacity of the ER (*right panel*), non-native proteins saturate the PDI substrate-binding site, resulting in the loss of Ero1α–PDI interaction. This inhibits both oxidative folding and the activation steps of Ero1α. Such inhibition would be independent of the thiol/disulfide status of the non-native protein. Inhibition of the activation steps occurs by both early-stage folding species and late-stage folding intermediates, whereas the Ero1α–PDI catalytic cycle is affected only by the early-stage folding species tested. Inhibition of activation, while inactivation continues, causes an accumulation of Ero1α in the inactive state. This dual action inhibition mechanism will prevent dithiol oxidation and keep client proteins in a more reduced state suitable for ERAD, allowing more efficient restoration of folding capacity.

In this study, we show that Ero1α–PDI interactions are indeed inhibited by unfolded proteins and by folding intermediates, as well as by peptides, endogenous molecules such as the hormone 17β-E2, and exogenous molecules such as BPA. Inhibition of the activated Ero1α–PDI redox cycle was achieved by a subset of molecules, including non-native proteins from early stages of the folding pathway, but not those tested from the late stages of the folding pathway. In contrast, all the tested inhibitors slowed down redox activation of Ero1α, indicating that the activation steps are especially vulnerable to competition for binding to the substrate-binding site of PDI. This suggests that inhibition of activation forms a primary route for reducing oxidative folding under conditions where the folding capacity of the ER has been exceeded (Fig. 7). It is important to note that this is not simply a case of when the folding capacity of PDI is exceeded, because other folding catalysts and molecular chaperones also bind to non-native proteins, increasing the threshold at which inhibition of Ero1 will be activated, and that this inhibition is independent of the presence/absence of cysteines or disulfides in the non-native proteins.

Inactivation mechanisms of Ero1α are still poorly understood. One unexpected result from our experiments was that the presence of a potent non-native protein inhibitor that compromised activation steps, but not the catalytic cycle (Fig. 2, *B* and *C*), resulted in a redox inactivation process for Ero1α that could be kinetically modeled (Fig. 2*D*). The half-time for this inactivation was ∼128 s, roughly equivalent to the activation kinetics. This suggests that there is a kinetic partitioning from the active state of Ero1α that can either lead to a new cycle of PDI oxidation or inactivation by formation of regulatory disulfide(s) (Fig. 7). With the 10-fold molar excess of PDI over Ero1α used in the experiments, the kinetics of the inactivation are ∼70-fold slower than the catalytic cycle (*k*_cat_ (0.39 s^−1^)/*k*_inactivation_ (0.0055 s^−1^)), suggesting that ∼1.4% of Ero1α gets inactivated per catalytic cycle. The kinetics of the inactivation for Ero1α has not, to our knowledge, been previously reported. These results also suggest that either the inactivation is catalyzed by PDI independently of the substrate-binding site or auto-oxidation of the Ero1α regulatory disulfides plays a role in the inactivation mechanisms.

Physiologically, these results have implications for the cross-talk between oxidative protein folding and UPR and ERAD. During basal protein synthesis, there is a balance between the load from the non-native proteins and the folding capacity provided by folding assistants. In this state, PDI, with local ER concentrations potentially reaching millimolar levels (based on quantification of total PDI from liver tissue (27, 28) and that ER constitutes <5% of total cellular volume (29)), is likely to be in excess over all non-native proteins leading to (partial) activation of Ero1α and an active oxidative protein folding cycle (Fig. 7, *left panel*). When non-native proteins accumulate in the ER and surpass a critical threshold, PDI no longer activates the inactive pool of Ero1α, whereas the active pool undergoes redox inactivation (Fig. 7, *right panel*). This prevents futile cycles of oxidation–reduction of non-native proteins, whereas the folding capacity of the ER is exceeded, saving cellular resources such as molecular oxygen. In addition, it will reduce the burden on the reductive component of ERAD, potentially increasing the efficiency of ERAD and decreasing the dependence on a separate reductase for the process. Oxidative protein folding can then be restored once the inhibiting non-native proteins are either directly removed from the system or sequestered by UPR up-regulation of folding factors, which leads to an increase in the level of free PDI with an unoccupied substrate-binding site able to reactivate Ero1α.

## Materials and methods

### Reagents

Bisphenol A, β-estradiol, and α-estradiol (Sigma–Aldrich) were dissolved in absolute ethanol to final concentrations of 100 mm and stored at −20 °C. The preparation of KFWWFS has been described (21), and the lyophilized peptide was dissolved in 10 mm HCl to a final concentration of 2.8 mm and stored at −20 °C. The β-hairpin of Ero1α (RYLLQETWLEKKWGH) with an N-terminal biotin label was synthesized by GenScript and dissolved at 0.02 μg/ml in binding buffer (20 mm sodium phosphate, pH 7.0, 300 mm NaCl) freshly before use. Catalase from bovine liver and NADPH (Sigma–Aldrich) were dissolved in 20 mm sodium phosphate, pH 7.0, 150 mm NaCl to final concentration of 1 unit/μl and 50 mm, respectively, and frozen as aliquots at −20 °C. GSH reductase from baker's yeast (Sigma–Aldrich) were freshly prepared before the measurements from an ammonium sulfate suspension by spinning down and resuspending to 20 mm sodium phosphate, pH 7.0, 150 mm NaCl to a final concentration of 0.05 unit/μl. Reduced GSH (Sigma–Aldrich) was dissolved in 20 mm sodium phosphate, pH was adjusted to 7.0 with NaOH, NaCl was added to 150 mm, and aliquots were stored at −20 °C.

### Molecular biology

Expression vectors encoding mature nontagged WT BPTI (Arg^36^–Ala^93^), as well as mature WT BPTI and BPTI mutants with an N-terminal His tag have been described previously (30, 31). From these vectors, His-tagged BPTI variants were subcloned into a polycistronic vector co-expressing mature, codon optimized yeast Erv1p and human PDI (32). All generated plasmids were verified by sequencing (Biocenter Oulu Core Facility).

### Protein expression, purification, and analysis

His-tagged mature PDI, Ero1α–PDI complex, Ero1α C166A, and Erv1p were expressed and purified as described (12). Unfolded reduced BPTI was expressed in inclusion bodies, purified, lyophilized, and resuspended to 10 mm HCl to 2.3 mm as described (30) with the exception of cultivating the strain in an autoinduction medium with a terrific broth base including trace elements (Formedium) and 0.8% (w/v) glycerol instead of LB. Folded His-tagged WT BPTI and the C14A/C38A and C30A/C51A mutants were expressed from polycistronic vectors also encoding Erv1p and PDI as folding factors as the Ero1α–PDI complex (12). The cells were pelleted, resuspended in 20 mm sodium phosphate, pH 7.3, 100 μg/ml lysozyme, 20 μg/ml DNase, and lysed by two rounds of freeze-thawing. The BPTI products were then purified by immobilized metal affinity chromatography and ion exchange as described (33) except with the latter step carried out in the same buffer in a 5-ml SP FF cation exchanger (GE Healthcare) and finally by size-exclusion chromatography equilibrated with 20 mm sodium phosphate, 150 mm NaCl, pH 7.0. The purity of the BPTI variants was confirmed by SDS-PAGE, and pure fractions were pooled, concentrated using Amicon Ultra centrifugal filters (3-kDa molecular mass cutoff), aliquoted, flash-frozen in liquid nitrogen, and stored at −70 °C.

Protein concentrations were determined spectrophotometrically at 280 nm using calculated molecular masses and absorption coefficients: PDI, 56379 Da, 45,755 m^−1^ cm^−1^; nontagged WT BPTI, 6649 Da, 5960 m^−1^ cm^−1^; His-tagged WT BPTI, 7603 Da, 6335 m^−1^ cm^−1^; and His-tagged BPTI Cys-to-Ala mutants, 7539 Da, 6210 m^−1^ cm^−1^. Determination of [Ero1α–PDI complex] and [Erv1p] by a method that corrects for the bound FAD contribution is described in Ref. 12. The molecular weight of purified proteins and the folding status of the BPTI variants (Table 1) were confirmed by electrospray ionization MS.

**Table 1:** **Major products of mass spectrometric analysis (Q Exactive Plus Biopharma Orbitrap) of purified BPTI variants compared to theoretical masses obtained from ProtParam tool** Gluconoylation of the N-terminal His tag (34) was also observed, but the disulfide status of these species was similar to the nongluconoylated species. These data indicate that the folded states contain the correct disulfides and the unfolded species is reduced.

Variant	Expected mass	Experimental mass	Δ_mass_	Explanation
	*Da*	*Da*	*Da*	
Unfolded reduced nontagged wildtype BPTI	6648.7	6648.3	−0.4	No disulfides
Folded His-tagged wildtype BPTI	7602.8	7596.4	−6.4	Three disulfides (−6 Da)
Folded His-tagged BPTI C14A/C38A	7538.7	7534.4	−4.3	Two disulfides (−4 Da)
Folded His-tagged BPTI C30A/C51A	7538.7	7534.5	−4.3	Two disulfides (−4 Da)

### Oxygen consumption assays

Activity of Ero1α and Erv1p was assessed by oxygen consumption measurements, collecting three to five activity traces per reaction condition. Because yields of protein were low, three batches of protein were used to produce the results presented. No batch-specific variance was seen. The activity traces and kinetic parameters *k*_cat_, half-time of activation, and Hill coefficient were obtained with the methods and conditions described in Ref. 12. Briefly, 1 μm of Ero1α–PDI complex or Erv1p were injected into a substrate solution containing 10 μm PDI, 10 mm GSH, GSH reductase, 1 mm NADPH, and, additionally, 10 units of catalase in 20 mm sodium phosphate, pH 7.0, 150 mm NaCl, 2 mm EDTA. The control reaction without PDI or inhibitors was collected similarly but injecting 1 μm of Ero1α C166A mutant instead of the Ero1α–PDI complex.

Inhibitors were added at 50 μm (unless otherwise indicated) either before Ero1α was injected to the assay, or they were injected at ∼50% oxygen saturation, *i.e.* after Ero1α had fully activated. Injection volumes of inhibitors were kept to less than 20 μl to avoid perturbations. For the KFWWFS titration, all reactions contain the same concentration of HCl that originates from the highest concentration of peptide used.

### Binding analysis

The isothermal titration calorimetry was performed using a MicroCal iTC-200 (Malvern) instrument. Both, PDI and KFWWFS peptide were diluted with 20 mm sodium phosphate, pH 7.0, 0.15 m NaCl to final concentrations of 27 and 600 μm, respectively, and treated with 1 mm DTT for 5 min at room temperature before the measurements. The titration was carried out at 25 °C with an initial 0.4-μl injection followed by 2.44-μl injections of KFWWFS to PDI every 180 s with a stirring rate of 750 rpm. The data were analyzed using ORIGIN software (OriginLab). The first injection was excluded from integration according to the manufacturer's instructions. The calculated stoichiometry of binding was 0.97:1.

BLI analysis was performed using an Octet Red 384 instrument (FortéBio Inc.) at 30 °C in binding buffer. KFWWFS (10 μg/ml) or the biotin-labeled β-hairpin of Ero1α (0.02 μg/ml) were immobilized onto AR2G or SA biosensors (FortéBio Inc.), respectively, according to the instructions from the manufacturer. Binding to KFWWFS was measured at 0, 0.5, 1.0, and 2.0 μm [PDI], and binding to β-hairpin was measured at 0, 0.5, 1.0, 2.0, and 4.0 μm [PDI]. For the direct competition assay, binding to β-hairpin was measured at 4.0 μm [PDI] with 20 μm KFWWFS present. All binding assays with β-hairpin had also 1 mm DTT. Association was collected for 5 min followed by 10 min of dissociation. The binding curves were fitted with a 1:1 binding model individually and separately in association and dissociation steps, and dissociation constant *K_d_* was calculated using the HT data analysis software (FortéBio Inc.). The measurements were collected at least in triplicate.

## Author contributions

A. M. formal analysis; A. M. investigation; A. M. methodology; A. M. writing-original draft; A. M. and L. W. R. writing-review and editing; L. W. R. conceptualization; L. W. R. supervision; L. W. R. funding acquisition.

## Footnotes

2 The abbreviations used are:

ER: endoplasmic reticulum
BLI: bilayer interferometry
UPR: unfolded protein response
ERAD: ER-associated degradation
PDI: protein disulfide-isomerase
Ero1: ER oxidoreductin-1
BPTI: bovine pancreatic trypsin inhibitor
E2: estradiol
BPA: bisphenol A.
